# Identification and analysis of extrachromosomal circular DNAs in pancreatic islets during the early and late stages of T2DM mice

**DOI:** 10.1016/j.gendis.2025.101914

**Published:** 2025-10-31

**Authors:** Zhichao Li, Yue Sun, Shujun Wan, Hongwen Chu, Deguo Wang, Kun Lv, Xiang Kong, Xinming Yao

**Affiliations:** a Anhui Provincial Key Laboratory of Non-coding RNA Basic and Clinical Transformation, Wannan Medical College, Wuhu, Anhui 241002, China; b Department of Gerontology, Geriatric Endocrinology Unit, The First Affiliated Hospital of Wannan Medical College, Wuhu, Anhui 241001, China; c National Clinical Research Center for Geriatric Diseases, Anhui Provincial Sub-center, Wuhu, Anhui 241001, China; d Department of Gerontology, The People's Hospital of Chizhou, Chizhou, Anhui 247000, China

Extrachromosomal circular DNAs (eccDNAs) have been implicated in the pathogenesis of various diseases, particularly in cancer, where they contribute to gene amplification and oncogene expression. However, the regulatory mechanisms of eccDNAs in type 2 diabetes mellitus (T2DM) during both the early and late stages remain unknown. Here, we employed circularization for *in vitro* reporting of cleavage effects by sequencing (CIRCLE-seq) to identify and analyze eccDNAs in pancreatic islets of T2DM mice at 8- and 24-week time points. As a result, the differentially expressed eccDNAs were 76 and 5422 in the 8- and 24-week groups, respectively. KEGG analysis showed that the glucagon signaling pathway was significantly enriched in the 8-week group. However, in the 24-week group, the pathways were primarily enriched in the phosphatidylinositol signaling system, the Wnt signaling pathway, pathways related to cancer, and the Rap1 signaling pathway. In particular, Wnt7a^circle^ and Braf^circle^ were significantly up-regulated, potentially contributing to cell proliferation and tumor development. The results of this study indicated that a substantial quantity of eccDNAs was generated during both stages and regulated various biological processes, suggesting that eccDNA accumulation may correlate with the elevated risk of cancer and the emergence of multiple complications in the later stages of T2DM.

T2DM is a complex metabolic disorder characterized by insulin resistance and impaired insulin secretion, resulting in elevated blood glucose levels.^1^ The heterogeneity of T2DM, along with genetic predispositions and environmental factors, contributes to the complexity surrounding its diagnosis and treatment.^2^ eccDNAs, which range in size from small non-coding repeats to large fragments containing functional genes, have been identified in a variety of eukaryotic organisms and human tissues.^3^ The biogenesis of eccDNA is dependent on several mechanisms, including rolling-circle replication, break-induced replication, and recombination events. Recent studies suggest that eccDNA may play a role in regulating gene expression, with the potential to influence disease pathogenesis, particularly in cancer.^4^ Interestingly, the presence of genes associated with metabolic regulation has been observed in eccDNAs, indicating their potential as novel diagnostic markers for T2DM and their potential impact on disease progression.^5^ To heighten the clinical relevance of our research, we employed genetically diabetic leptin receptor-mutated (db/db) mice as a model for T2DM and isolated pancreatic islets at both the early (8-week group) and late (24-week group) stages. Subsequently, CIRCLE-seq was utilized to identify differentially expressed eccDNAs, followed by functional analysis. The experimental setups of eccDNAs CIRCLE-seq are depicted in Figure 1A.

**Figure 1:**
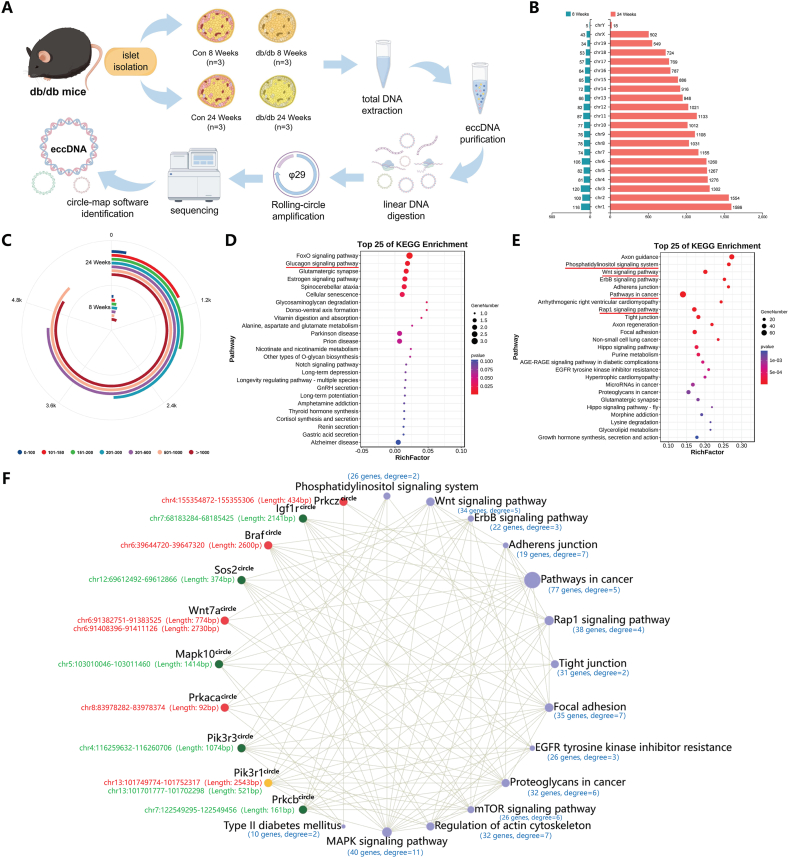
Characterization of eccDNAs in pancreatic islets of T2DM mice at 8 and 24 weeks. **(A)** The experimental setups of eccDNAs CIRCLE-seq. **(B)** Quantitative distribution of eccDNAs across different chromosomes. **(C)** Length-density distribution of eccDNAs. **(D)** KEGG enrichment analysis of origin genes of differentially expressed eccDNAs in the 8-week group. **(E)** KEGG enrichment analysis of origin genes of differentially expressed eccDNAs in the 24-week group. **(F)** Interaction network analysis of core eccDNAs with signal pathway in the 24-week group. The network diagram is labelled with the number of eccDNA origin genes enriched in signaling pathways and the degree of pathway connectivity. The chromosome locations of the eccDNA origin genes, along with the length information of the eccDNA, are also annotated. Purple dots represent signaling pathways, and the size of the dots indicates the number of eccDNA origin genes enriched in each pathway. Core eccDNAs are not distinguished by dot sizes; the red represents up-regulation, the green represents down-regulation, and the yellow indicates the presence of both up-regulated and down-regulated eccDNAs.

To detect eccDNA from similar pair regions of the mouse genome in pancreatic islets of T2DM mice at different disease stages, we used the Circle-Map software to identify eccDNAs under the screening condition with split reads ≥1. As a result, 1538 and 20,804 eccDNAs were found in pancreatic islet samples obtained from the 8- and 24-week groups, respectively. The distribution of eccDNAs on chromosomes is shown in Figure 1B, which revealed that the number of eccDNAs increased significantly in the later stages of T2DM (24-week group). Notably, all chromosomes had eccDNA distribution, and the number of eccDNAs exceeded 500 in all chromosomes except the Y chromosome. The length of eccDNAs in mouse pancreatic islets varied greatly, but was mainly distributed in regions larger than 200 bp, where in the 24-week group, eccDNAs with lengths in the range of 201–300 bp, 301–500 bp, 501–1000 bp, and >1000 bp were numbered as 2972, 4540, 5258, and 5022, respectively (Fig. 1C). The peak value of the GC content of eccDNAs was 41.94% ± 7.08% and 42.37% ± 6.99%, respectively, which were not significantly different compared with upstream regions, down-stream regions, and randomly generated *in silico* eccDNAs in the 8- and 24-week groups (Fig. S1A). The results of the motif characterization of the 10 bp sequence before and after the start and end sites of the eccDNAs are shown in Figure S1B and S1C. Interestingly, a repeating nucleotide pattern was identified between the start and end sites of ec-cDNAs in the 8- and 24-week groups, which may contribute to eccDNA formation.

Based on the screening conditions |log_2_ fold change| ≥ 1 and *p* < 0.05, followed by the application of Benjamini-Hochberg false discovery rate correction (*P*_*adj*_ < 0.05), we screened 76 and 5422 significantly dysregulated pancreatic islets eccDNAs in the 8- and 24-week groups, respectively. Of these, 21 were significantly up-regulated, 55 were significantly down-regulated, and 1462 had no significant change in the 8-week group (Fig. S2A); 2316 were significantly up-regulated, 3106 were significantly down-regulated, and 15,382 had no significant change in the 24-week group (Fig. S2C). To further investigate the biological functions of eccDNAs, we performed Gene Ontology (GO) and Kyoto Encyclopedia of Genes and Genomes (KEGG) enrichment analyses of eccNDAs' origin genes, including the identification of relevant cellular components, molecular functions, biological processes, and signaling pathways. According to the GO and KEGG analysis results, the 8-week and 24-week groups displayed different biological functions. In the 8-week group, the biological functions of eccDNA origin genes were mainly enriched in the regulation of peptidyl-lysine acetylation, regulation of intrinsic apoptotic signaling pathway in response to DNA damage, regulation of protein acetylation, and SCF ubiquitin ligase complex (Fig. S2B). The signaling pathways enriched in this group included the FOXO signaling pathway and the glucagon signaling pathway (Fig. 1D). In contrast, in the 24-week group, eccDNA origin genes were mainly enriched for axonogenesis, dendrite development, cell junction assembly, neuron to neuron synapse, synaptic membrane, postsynaptic specialization, cell adhesion molecule binding, PDZ domain binding, and actin binding (Fig. S2D). The major signaling pathways enriched in the 24-week group included the phosphatidylinositol signaling system, Wnt signaling pathway, and pathways in cancer (Fig. 1E).

Subsequently, network interactions were employed to elucidate the interaction relationship between core eccDNAs and signaling pathways in the 24-week group. The result revealed an interaction between 14 signaling pathways and 10 core eccDNA origin genes (Fig. 1F). The 14 signaling pathways include the MAPK signaling pathway, adherens junction, focal adhesion, regulation of actin cytoskeleton, proteo-glycans in cancer, mTOR signaling pathway, Wnt signaling pathway, pathways in cancer, Rap1 signaling pathway, ErbB signaling pathway, EGFR tyrosine kinase inhibitor resistance, phosphatidylinositol signaling system, tight junction, and type II diabetes mellitus. Among the 10 core eccDNAs, Prkcz^circle^, Braf^circle^, Wnt7a^circle^, and Prkaca^circle^ were up-regulated, while Igf1r^circle^, Sos2^circle^, Mapk10^circle^, Pik3r3^circle^, and Prkcb^circle^ were down-regulated. Notably, Pik3r1^circle^ exhibited both up-regulation and down-regulation in expression.

Finally, three eccDNAs with significantly elevated expression levels were randomly selected for validation of their circular characteristics through Sanger sequencing. Using the designed primers, PCR amplification products of Wnt7a^circle^, Pax3^circle^, and Foxo3^circle^ were successfully obtained, with sizes of 340 bp, 292 bp, and 336 bp, respectively (Fig. S3A). The base sequences at the junction sites for Wnt7a^circle^, Pax3^circle^, and Foxo3^circle^ were identified as GCTG, TCGG, and AACC, respectively, through Sanger sequencing analysis of the PCR products (Fig. S3B). In addition, we validated the eccDNA sequencing data by quantitative reverse transcription PCR (qRT-PCR) using outward-facing primers and by also assessing the mRNA expression levels of three up-regulated target genes (Fig. S3C and S3D; Table S1). These results confirmed that all three eccDNAs were successfully validated by Sanger sequencing (including their circular structure and junction site).

This study characterized eccDNAs and investigated the expression profile at different stages of T2DM, revealing significant variations in eccDNAs expression profiles across disease stages. In the early stage, eccDNAs primarily participated in insulin resistance processes, while in later stages, they became notably enriched in pathways associated with cell proliferation and tumorigenesis. These findings enhanced our understanding of T2DM progression and provided valuable insights for clinical interventions.

## CRediT authorship contribution statement

**Zhichao Li:** Writing – original draft, Data curation, Writing – review & editing, Software, Conceptualization. **Yue Sun:** Methodology, Data curation, Investigation. **Shujun Wan:** Methodology, Investigation. **Hongwen Chu:** Methodology, Data curation. **Deguo Wang:** Validation, Software. **Kun Lv:** Supervision. **Xiang Kong:** Writing – review & editing, Supervision, Writing – original draft. **Xinming Yao:** Visualization, Methodology, Formal analysis.

## Ethics declaration

The experimental protocols in this study obtained ethical approval from the Animal Ethics Committee of Wannan Medical College (WNMC-AWE-202290) and adhered to the Guide for the Care and Use of Laboratory Animals published by the National Institutes of Health (NIH Publication #85-23, revised 1996).

## Funding

This research was supported by the National Natural Science Foundation of China (81970699), Research Project of Distinguished Young Scholars of Universities in Anhui Province (2022AH020075), Anhui Provincial Natural Science Foundation (2308085MH252), Wuhu Science and Technology Project (2023jc27), Anhui Provincial Key R&D Program (2022e07020019), the Youth Health Research Project in Anhui Province (AHWJ2023A30185) and Scientific Research Foundation for the PhD (YR202452).

## Conflict of interests

The authors declared no competing interests.
