# Supplementary material for: Identification and analysis of extrachromosomal circular DNAs in pancreatic islets during the early and late stages of T2DM mice
Source: Genes Dis. 2025 Oct 31;13(3):101914. doi: 10.1016/j.gendis.2025.101914 (PMC12859182; doi:10.1016/j.gendis.2025.101914)
Supplement: Multimedia component 1 [file mmc1.docx]

**Identification and analysis of extrachromosomal circular DNAs in pancreatic islets during the early and late stages of T2DM mice**

**SUPPLEMENTARY DATA**

**1. Supplementary Materials & Methods**

**Experimental Animals**

The experimental protocols in this study obtained ethical approval from the Animal Ethics Committee of Wannan Medical College (WNMC-AWE-202290) and adhered to the Guide for the Care and Use of Laboratory Animals published by the National Institutes of Health (NIH Publication #85-23, revised 1996). Four-week-old male C57BKS-db/db mice (n=12) and their corresponding control C57BLKS/J (Con) mice (n=12) were purchased from GemPharmatech Co., Ltd (Nanjing, China). The animals were housed in a temperature-controlled room (22±4℃) with a consistent environment and a 12-hour light-dark cycle, while having unrestricted access to food and water. After one week of adaptation to feeding, the mice were divided into four groups: an 8-week control group and experimental group, as well as a 24-week control group and experimental group, each consisting of six mice. Throughout the study, blood glucose monitoring was conducted, and fasting blood glucose levels exceeding 11.1 mmol/L at 8-week-old indicated the development of T2DM in the experimental group mice.

**Isolation of mouse pancreas islets**

Mouse pancreas islets were isolated through collagenase digestion according to a previously described method [1]. Briefly, after euthanizing the mice through vertebral dislocation, the abdominal cavity was opened to fully expose the common bile duct and duodenum. The common bile duct was then carefully separated from the duodenal wall at its origin. Approximately 3 mL of collagenase P (Cat. No: 11213865001, Roche, Mannheim, Germany) at a concentration of 0.5 mg/mL was slowly infused into the common bile duct, gradually stopping the infusion when the tail of the pancreas was adequately filled with fluid and there was a noticeable increase in resistance. Once this was achieved, the pancreas was carefully removed without damaging the delicate islet cells. It was placed in 3 mL of collagenase solution and digested in a water bath at a constant temperature of 38±0.5℃ until most of the pancreatic tissue reached a consistency between chyle-like and sediment-like. To terminate the digestion, 35 mL of 4℃ Hank's solution was added to stop the digestion, rinse away the collagenase, and dis-perse the islets. Subsequently, pancreatic tissues were collected for circle-seq. To ensure sufficient material for circle-seq analysis, the pancreatic islets from two mice were pooled into a single sample.

**Circle sequencing**

Circle-seq was employed to identify pancreas islets eccDNAs in 8- and 24-week groups. The pancreatic islets were suspended in L1 solution (Plasmid Mini AX; A&A Biotechnology) and supplemented with Proteinase K (Thermo Fisher), followed by overnight incubation at 50 ℃ with agitation. After lysis, alkaline treatment, protein precipitation, and separation of chromosomal DNA from circular DNA were per-formed using an ion exchange membrane column (Plasmid Mini AX; A&A Biotechnology). The purified DNA obtained through the column was then treated with Fast Digest MssI (Thermo Scientifific) at 37 ℃ for 16 hours to eliminate mitochondrial circular DNA. Exonuclease digestion using Plasmid-Safe ATP-dependent DNase (Epicentre) was carried out at 37 ℃ in a heating block to remove any remaining linear DNA. The enzyme reaction continued for one week, with additional ATP and DNase added every 24 hours (30 units per day), following the manufacturer's protocol for Plasmid-Safe ATP-dependent DNase (Epicentre). Samples enriched in eccDNAs served as templates for phi29 polymerase amplification reactions, conducted at 30 ℃ over a period of two days (46-48 hours) using the REPLI-g Midi Kit. The resulting Phi29-amplified DNA was fragmented by sonication using a Bioruptor device, and the fragmented DNA underwent library preparation utilizing the NEBNext® Ultra II DNA Library Prep Kit for Illumina provided by New England Biolabs. Sequencing was carried out on Illumina NovaSeq with 150bp paired end mode.

**GO and KEGG analysis**

The paired-end reads were obtained from the Illumina NovaSeq 6000 sequencer and subjected to quality control using Q30 criteria. Subsequently, the cutadapt software (v1.9.1) was employed for 3' adaptor-trimming and removal of low-quality reads. The resulting high-quality clean reads were then aligned to the reference genome (UCSC GRCm39) using bwa software v (v0.7.12). For the detection of eccDNAs in all pancreatic islet samples, the circle-map software (v1.1.4) was employed, while samtools (v0.2) software was utilized to obtain raw soft-clipped read counts at the breakpoint. A com-prehensive statistical analysis was performed to investigate the abundance, length distribution, GC content, as well as motif characteristics of eccDNAs. Normalization and differential expression analysis of eccDNAs were performed using edgeR (v0.6.9) software, with a filtering criterion of |log2 Fold Change| ≥ 1 and P-value < 0.05 applied. Annotation of the eccDNAs was conducted using Bedtools (v2.27.1) software. The gene from differentially expressed eccDNA were subjected to Gene Ontology (GO) and Kyoto Encyclopedia of Genes and Genomes (KEGG) pathway enrichment analyses.

**Interaction network analysis**

To further understand the relationship between the signaling pathways and differ-ent expression eccDNAs in the 24-week group, we performed an interaction network analysis by integrating the results of KEGG enrichment analysis with the origin genes of the differentially expressed eccDNAs. Signaling pathways with a degree ≥ 2 and FDR < 0.05 were selected to construct an interaction network with the core eccDNAs. In the network diagram, purple dots represent signaling pathways, and the size of the dots indicates the number of eccDNA origin genes enriched in each pathway. Core eccDNAs were not distinguished by dot size; instead, red represents up-regulation, green represents down-regulation, and yellow represents the presence of both up- and down-regulated eccDNAs. The KEGG enrichment pathways analysis and interaction network analyses were conducting using OmicShare cloud (https://www.omicshare.com/).

**sanger sequencing and qRT-PCR verification**

Validation of three markedly upregulated eccDNAs from distinct gene regions was performed using Sanger sequencing. Initially, total DNA was extracted from pancreas islets samples and subjected to treatment with FastDigest MssI and exonuclease for the removal of mitochondrial circular DNA and linear DNA. Rolling circle amplification was subsequently implemented to increase the yield. PCR was performed using Accu-rate Taq Master Mix (dye plus) from Accurate Biotechnology in China to assess the expression levels of potential eccDNAs. The reaction parameters included an initial denaturation step at 94 ℃ for 30 seconds, followed by 28 cycles of denaturation at 98 ℃ for 10 seconds, annealing at 55 ℃ for 30 seconds, and extension at 72 ℃ for one mi-nute. This was followed by a final elongation step at 72 ℃ for two minutes and stor-age at 4 ℃. The primers designed for amplifying the eccDNAs were based on the "out-facing" approach and are detailed in Table 1.

Subsequently, the PCR products were loaded onto 1.5% agarose gels and visualized under an ultraviolet Luminescent Image Analyzer (LAS-4000 Mini; GE Healthcare Life Sciences, Pittsburgh, USA). Positive bands indicating specific amplification were purified and subjected to TOPO-TA cloning (Zero TOPO-TA Cloning Kit from Shanghai Yeasan, China), followed by Sanger sequencing analysis (performed by Shanghai San-gon Biotech in China). A comparison between Sanger sequencing results and high-throughput sequencing data was conducted to assess any discrepancies in nucleotide composition among each positive PCR product. In addition, The CT values of the three target eccDNA and PGEX-5X-2 were calculated and the relative expression levels of eccDNA were analyzed using the 2^−ΔΔCt^ method. The detailed steps of qRT-PCR were referred to in our previously published article[2].

**Statistical analysis**

Student’s t test was performed for the comparison of two groups. Data from more than two groups were compared using one-way ANOVA followed by Tukey’s multiple comparison tests. Data are presented as means ± SD.

**2. Supplementary Figures**

**
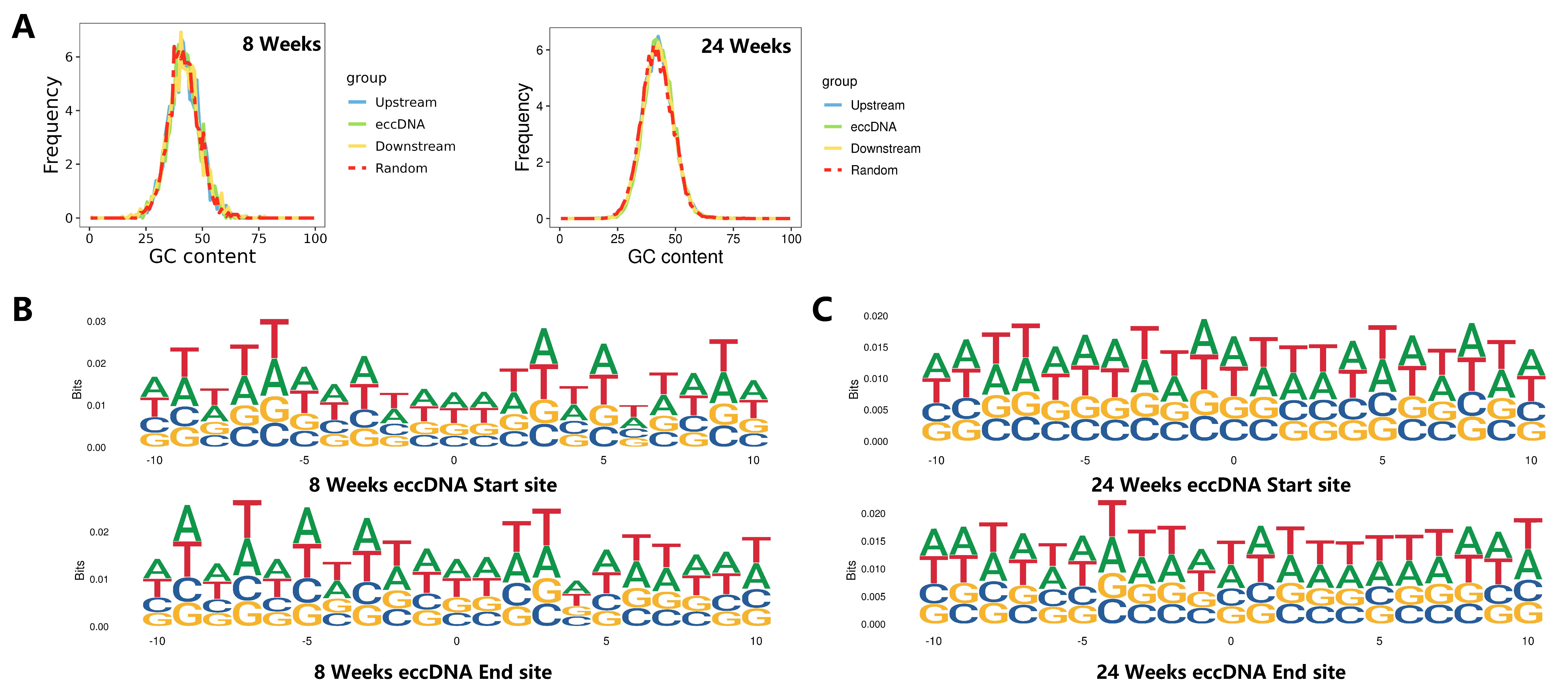
Figure S1. Characteristics of eccDNAs distribution in pancreatic islets of T2DM mice at 8- and 24-week groups.** (A) Distribution of GC contents in eccDNAs. (B) Motif characteristics of the 10 bp sequence before and after the start and end sites of eccDNAs in the 8-week group. (C) Motif characteristics of the 10 bp sequence before and after the start and end sites of eccDNAs in the 24-week group.

**
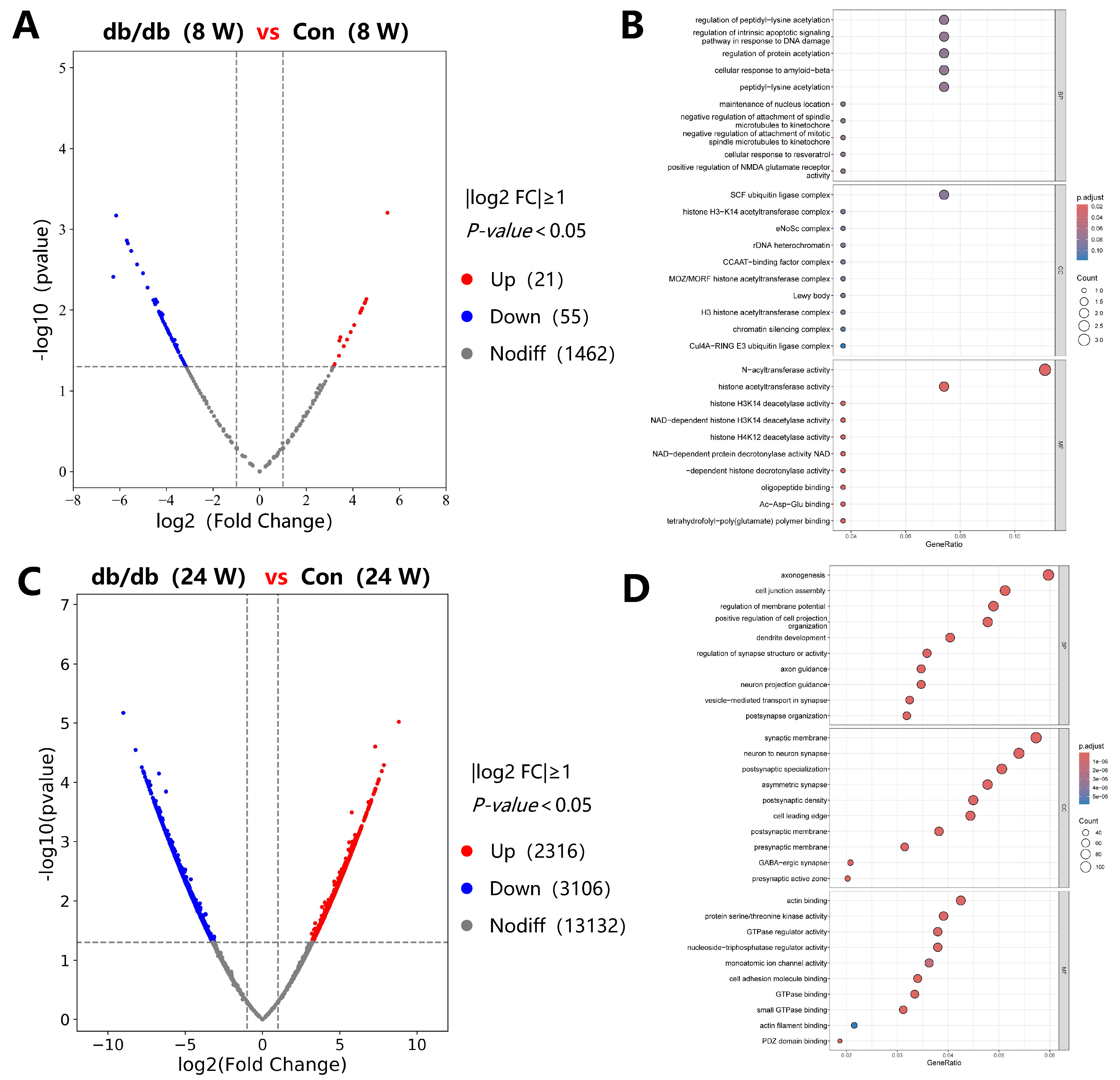
Figure S2. Counting of different expression eccDNAs in pancreatic islets of T2DM mice at 8- and 24-week groups and functional enrichment of eccDNA origin genes using GO.** (A) Volcano plot displaying differentially expressed eccDNAs in the 8-week group. (B) GO enrichment analysis of origin genes of different expression eccDNAs in the 8-week group. (C) Volcano plot displaying differentially expressed eccDNAs in the 24-week group. (D) GO enrichment analysis of origin genes of differentially expressed eccDNAs in the 24-week group.

**
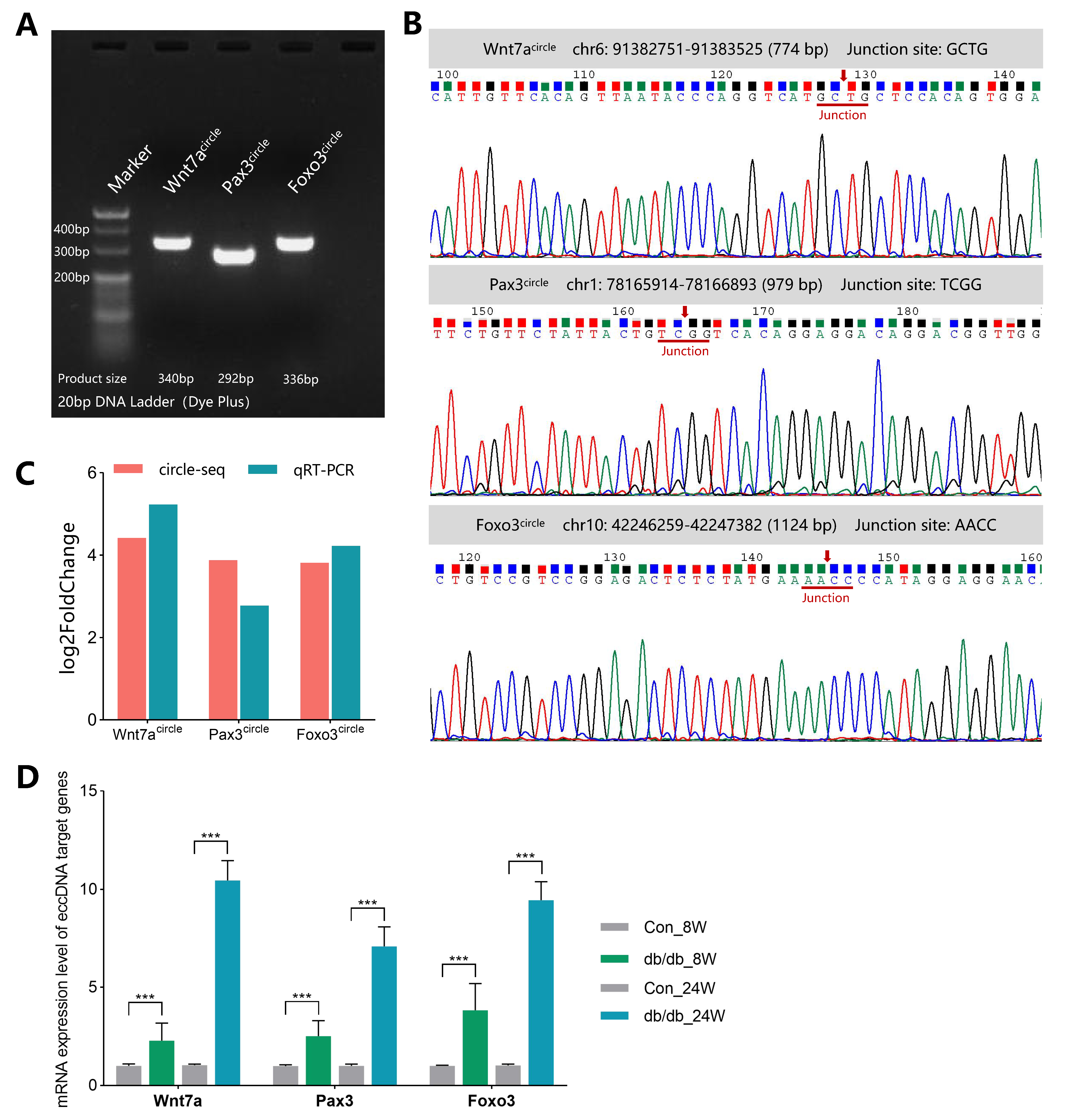
Figure S3. Validation of the eccDNAs in the pancreatic islets.** (A) PCR amplification products of three eccDNAs. (B) Junction sites obtained after sanger sequencing of PCR products. (C) Three up-regulated eccDNAs were selected to validate using qRT-PCR with the outward facing primers. (D) The mRNA expression level of three up-regulated eccDNA target genes.

Table S1. Paired primers for qPCR of genes

| Gene | Accession no. | Forward Primer | Reverse Primer |
| --- | --- | --- | --- |
| *β-Actin* | NM_007393.5 | TGCTGTCCCTGTATGCCTCT | TTGATGTCACGCACGATTTC |
| *Wnt7a* | NM_001363757.1 | CTCTTTCTCAGCCTGGGCAT | GAGCCTTCTCCTATGACGATGA |
| *Pax3* | NM_001159520.1 | CAAACCCAAGCAGGTGACA | AGGATGCGGCTGATAGAACTC |
| *Foxo3* | NM_001376967.1 | TGTCACACTACGGCAACCAG | GGTGGAGCAAGTTCTGATTGA |

**Author Contributions**

Conceptualization, Xiang Kong and Xinming Yao; methodology, Zhichao Li.; software, Zhichao Li and Yue Sun.; validation, Shujun Wan, Hongwen Chu and Deguo Wang; formal analysis, Kun Lv; investigation, Zhichao Li and Yue Sun; resources, Xiang Kong; data curation, Shujun Wan and Hongwen Chu; writing-original draft preparation, Zhichao Li; writing-review and editing, Zhichao Li and Xiang Kong; visualization, Xinming Yao; supervision, Kun Lv; project administration, Zhichao Li; funding acquisition, Xiang Kong and Xinming Yao. All authors have read and agreed to the published version of the manuscript.

**Funding**

This research was supported by the National Natural Science Foundation of China (81970699), the Research Project of Distinguished Young Scholars of Universities in Anhui Province (2022AH020075), the Anhui Provincial Natural Science Foundation (2308085MH252), the Wuhu Science and Technology Project (2023jc27), the Anhui Provincial Key R&D Program (2022e07020019), the Youth Health Research Project in Anhui Province (AHWJ2023A30185) and Scientific Research Foundation for the PhD (YR202452).

**Informed Consent Statement**

Not applicable.

**Data Availability Statement**

Data will be made available on request. The Circle-seq data have been deposited into the Genome Sequence Archive (https://ngdc.cncb.ac.cn/gsa/) with accession number PRJCA031039.

**Conflicts of Interest**

The authors declare that they have no conflict of interest. The funders had no role in study design, data collection and interpretation or in the decision to submit the work for publication.

**Abbreviations**

Sirt1^circle^ Sirtuin 1^circle^

Prkcz^circle^ Protein kinase C zeta^circle^

Braf^circle^  Braf transforming gene^circle^

Wnt7a^circle^ Wingless-type MMTV integration site family member 7A^circle^

Prkaca^circle^ Protein kinase alpha^circle^

Igf1r^circle^ Insulin-like growth factor I receptor^circle^

Sos2^circle^ Son of sevenless homolog 2^circle^

Mapk10^circle^ Mitogen-activated protein kinase 10^circle^

Pik3r3^circle^ Phosphatidylinositol 3 kinase 3^circle^

Prkcb^circle^ Protein kinase C beta^circle^

Pik3r1^circle^ Phosphatidylinositol 3 kinase 1^circle^

**References:**

[1]. Sun, Y., et al., Metformin alleviates glucolipotoxicity-induced pancreatic beta cell ferroptosis through regulation of the GPX4/ACSL4 axis. Eur J Pharmacol, 2023. 956: p. 175967.

[2]. Kong, X., et al., Increased serum extrachromosomal circular DNA SORBS1(circle) level is associated with insulin resistance in patients with newly diagnosed type 2 diabetes mellitus. Cell Mol Biol Lett, 2024. 29(1): p. 12.
